# InChI - the worldwide chemical structure identifier standard

**DOI:** 10.1186/1758-2946-5-7

**Published:** 2013-01-24

**Authors:** Stephen Heller, Alan McNaught, Stephen Stein, Dmitrii Tchekhovskoi, Igor Pletnev

**Affiliations:** 1 Department of Chemistry, InChI Trust, InChI Trust, NIST, NIST, Lomonosov Moscow State University, Moscow, Russia

## Abstract

Since its public introduction in 2005 the IUPAC InChI chemical structure identifier standard has become the international, worldwide standard for defined chemical structures. This article will describe the extensive use and dissemination of the InChI and InChIKey structure representations by and for the world-wide chemistry community, the chemical information community, and major publishers and disseminators of chemical and related scientific offerings in manuscripts and databases.

## Background and history

While molecular species with known structures have been the building blocks of chemistry since the mid-19th century, representing and communicating the structural information has always been challenging, requiring highly specialized knowledge and training. The multiple ways of representing a molecule and the different levels of uncertainty regarding this representation have been a central part of this expertise. However, the number of uses and known structures has exploded over the past 50 years, resulting in an information overload unmanageable to many organizations. Only with the advent of computers, capable of providing digital representations and software for handling them, was there seen to be a means of dealing with this problem. Since the 1960s, when the development of computerized structure representations first became a practical possibility, there has been a need for a uniform method to perform this function. From the 1960s to the end of the 20th century many organizations developed structure representations, most of which were used just for in-house/internal needs or for computer-based commercial products. These include WLN, DARC/ELCO, GREMAS, Hayward, SMILES, ROSDAL, SMD, Molfile, and CAS Registry [1-3]. As computer systems, both hardware and software, developed and expanded in size and capability most of these representations died out and only a few remained in widespread use: namely SMILES, CAS, and Molfiles in SDfiles [4]. SMILES was designed for and primarily used for in-house databases. The CAS system was designed for and used primarily for the Chemical Abstracts database [5]. Computer-based chemistry resources added SMILES representations and/or CAS Registry Numbers to their products or information; this was, and is, of use to those in need of such facilities. Use of either of these systems required close cooperation with third parties and made compound ‘registrations’ complex and expensive owing to the involvement of a commercial organization. The problem with both these representations was that, being proprietary, their ability to be used world-wide by anyone with a source linked to information and data on a chemical was and still is, extremely limited. While this situation could have gone on for a long time, the status quo was upset by a ‘black swan’ event [6], the appearance of the Internet. For the last few decades of the 20th century most chemical information was made available electronically, either in-house or online; the Internet had changed everything. Chemists throughout the world were now able to access a vast amount of information, both free and fee-based. This enabled providers to greatly expand their markets, which led to increased usage (and revenue where fee-based), but also led to the obvious need for these many resources to be better linked than they had been in the past - and the realization that this now could be easily done and the information readily accessed. At the same time the Internet was creating another black-swan or transformative event – the development of Open Source computer code. This combination of the Internet, Open Source, the realization that virtually all chemical information could be linked, and the need for a, computer-based alternative to IUPAC’s lengthy and complex chemical nomenclature rules provided the perfect (good) storm for the creation of the IUPAC InChI standard.

Added to this perfect storm was the need for the various physical chemical databases at the US standards agency, NIST, to have the same structure representation for NIST databases and for in-house quality control procedures [7]. Had the decision to proceed on this project been made in 1990 rather than 2000 the work would have progressed as an excellent and useful internal project, no doubt written up and published as yet another example of a way to represent chemical structures. Addition of this new participant, NIST, combined with ongoing IUPAC involvement in the development of systematic and standard procedures for naming chemical substances on the basis of their structure, meant that all the pieces were in place for the InChI project to be initiated.

In March 2000 IUPAC convened a meeting in Washington, DC to look into the matter of chemical structure representation [8]. The IUPAC Strategy Roundtable meeting was called “Representations of Molecular Structure: Nomenclature and its Alternatives”. It brought together 41 participants from 10 countries including experts in organic, inorganic, biochemical, and macromolecular nomenclature; users of nomenclature in academia, industry, patents, international trade, health and safety communities; journal editors and publishers; database providers; and software vendors.

As mentioned above, with the ever-increasing reliance on computer processing by chemists, it became evident to many within IUPAC that this organization should find better ways of handling nomenclature than was done in the past. In particular it was felt by the authors of the present paper that while IUPAC had stressed conventional chemical names/nomenclature in the 20th century, continued progress into the 21st century required new, computer-driven approaches to the problem of chemical identification.

At the meeting in March 2000 three of the authors of this article [Stephen Heller (SRH), Stephen Stein (SES), & Dmitrii Tchekhovskoi (DT)] presented a proposal to IUPAC, which extended one developed by one of the authors (SRH) in the fall of 1999. The initial proposal from November 1999 had been widely circulated within the chemical information and chemical structure representation community via e-mail. The proposal presented at the March 2000 meeting incorporated considerable improvements from feedback from chemists in the USA, Europe, and Asia. At the end of the March 2000 meeting Bill Town [9] proposed that the new program be called IUPAC Chemical Identifier Project (IChIP).

The aim of the IUPAC Chemical Identifier Project (IChIP) was to establish a unique label, the IUPAC International Chemical Identifier (InChI), which would be a non-proprietary identifier for chemical substances that could be used in printed and electronic data sources thus enabling easier linking of diverse data compilations and unambiguous identification of chemical substances.

It was agreed at that time that InChI would not be a registry system. It would not depend on the existence of a database of unique substance records to establish the next available sequence number for any new chemical substance being assigned an InChI. It would be based on a set of IUPAC structure conventions, and rules for normalization and canonicalization (1c) of an input structure representation to establish the unique label. It would thus enable an automatic conversion of a graphical representation of a chemical substance into the unique InChI label which could be created independently of any organization anywhere in the world and which could be built into any chemical structure drawing program and created from any existing collection of chemical structures.

As a result of the meeting and the recommendations in the report [8] the following scheme was approved by IUPAC in April 2000 [10]:

1. An ad hoc Committee on Chemical Identity and Nomenclature Systems (CCINS) was established, with Alan McNaught (ADM), who at the time was at the Royal Society of Chemistry in Cambridge UK, as Chairman. The CCINS was responsible for developing systems for conventional and computer-based chemical nomenclature; cooperating with the four current IUPAC Nomenclature Commissions; coordinating interdisciplinary activities in the nomenclature field; and recommending to the IUPAC long-range strategy on chemical nomenclature. It was expected that this body would provide the long-term central planning, management and coordination of chemical nomenclature that would otherwise be lost when the Commissions were discontinued at the end of 2001.

2. A feasibility study of the Chemical Identifier project, to be managed by the CCINS, was initiated. A “chemical identifier” was intended to be a meaningful alphanumeric text string that could uniquely identify a chemical compound and facilitate its handling in computer databases. This code would be the equivalent of an IUPAC systematic name and would be designed to provide information about the specific substance in question. Since there were several issues to be resolved, the participants in the Nomenclature Round Table recommended that the feasibility of the project and resolution of these issues be carried out as soon as possible by representatives of a wide range of interested parties. Stephen Heller (SRH) and Stephen Stein (SES) (NIST) were asked to recommend a list of individuals and groups that should be consulted initially and to propose a framework for addressing the issues.

In August 2000 a meeting was held in Cambridge UK to discuss a number of technical issues before work on the project began. A detailed proposal was then prepared and IUPAC requested assistance from NIST to provide the bulk of the technical support for the project. In December 2000 the project was approved by IUPAC and officially started on January 1, 2001 [11]. Initial reports were presented at the IUPAC 38th Congress – (an invited talk on the IUPAC InChI Project) in July, 2001, at the ACS National Meeting in Chicago, Illinois in August, 2001, at the CAS/IUPAC Conference on Chemical Identifiers and XML for Chemistry in July, 2002, and at the US Government Conference on Chemical Databases in July, 2003 [12]. In addition, a number of articles regarding the InChI project appeared in chemistry and other science publications [13,14].

### Subcommittee on the IUPAC chemical identifier (InChI)

The ad hoc Committee on Chemical Identity and Nomenclature Systems (CCINS) was eventually replaced by the new IUPAC Division VIII (Chemical Nomenclature and Structure Representation Division) and responsibility for the InChI project was given to the Division VIII Subcommittee on the IUPAC Chemical Identifier (InChI) [15] where it currently resides [16]. Two of the authors of this article, the chairman (SRH) and secretary (ADM) of the IUPAC subcommittee have been in their positions from the start of the subcommittee activities. The subcommittee and its subsidiary working groups consist of a few dozen chemists and scientists with appropriate expertise; the numerous working groups handle specific areas of chemical structure representation. It is the responsibility of the subcommittee to approve all structure representation standards, which are then implemented by programmers. The usual IUPAC process of review has been amended for this project so that the work can proceed with the speed necessary for the scientific community to accept and use the results.

### IUPAC InChI working groups

While the IUPAC InChI subcommittee has overall technical responsibility for approving the structure standards recommended, it is the individual working groups, appointed by the subcommittee, that actually do the work to develop the structure standards. Since the inception of the subcommittee, the following groups have been created, a number of which have completed their tasks. In general each working group consists of six to twelve experts in the area under discussion, and works primarily via email, but does also have one or more face-to-face meetings over the lifetime (1–2 years) of the group. It was, and still is believed that by appointing acknowledged experts and leaving them free to make the final recommendation on what the standard will be has been critical to the success of the project. Confidence that what one recommends as a standard will really be accepted has assured the dedication to and success of the project.

The following is a list of working parties and their areas of chemistry or related InChI needs.

Inorganics

Organometallics

Polymers & Mixtures

InChI for Chemical Reactions

InChI Resolver

Markush (generic structures found in patents – see reference [17]

Electronic/Excited States

InChI for Materials Science

Redesign for handling tautomerism

Revised InChI FAQs

InChI Certification Suite

As one can see from the above list, there are still areas that need to be covered, such as alloys, phase diagrams, rotaxanes, and so on.

### InChI trust

As the IUPAC InChI structure standard began to take hold there was concern amongst a number of people involved in the project as to what would happen to the algorithm that had been developed at NIST. IUPAC is essentially an all-volunteer organization, so a mechanism needed to be found to assure the long term viability of the algorithm. It will be a surprise to no one involved in trying to develop a standard that it is easy to state the goal and create a standard, but rather difficult to actually implement it and have it accepted and used. The three main reasons in the present case are financial support, agreement of a critical mass of potential users to use the algorithm, and agreement that it will be the standard.

While having the blessing and support of IUPAC is certainly a necessary condition for InChI to become the chemical structure standard for the world-wide chemistry and the wider scientific communities it is by no means sufficient. Many excellent technical standards have had stronger and more potent blessings, yet have failed to take hold. For example, we all know that the metric system is used virtually throughout the world, save the United States [18]. For whatever reasons the US Government has yet to endorse or support the metric system, or to move forward to make it a standard in the country with the world’s largest economy. The Metric Act of 1866 (signed by the President on July 27, 1866), also known as the Kasson Act, has yet to be implemented, accepted, or widely used. On December 23, 1975 the Metric Conversion Act, Public Law 94–168 was passed. Furthermore, on August 9, 2007, the Act was amended by Public Law 110–69, as the America COMPETES Act. Among other things it replaced the old (1866) definition of the metric system with the modern-day definition of SI. Yet, today one finds virtually no sign of the metric system being used in the USA, save a few highway speed limit signs near the US-Mexico border.

Those managing the InChI project understood that success of the InChI algorithm as a standard, required its adoption beyond NIST, in particular by established commercial companies convinced of its value and in a position to provide financial support. Meetings were arranged with publishers, database producers, and software vendors; all saw the value of having a chemical structure standard that would make their products more visible and available to their community of users. And since all of these commercial organizations saw InChI as a pre-competitive activity, there was little problem in getting the many parties together to agree to use the InChI algorithm, and to agree also that it would be the standard. Supporting their decision was the realization that the other two widely used structure identifiers, CAS Registry Numbers and SMILES, could never be widely adopted owing to their being proprietary and/or too costly for acceptance by thousands of sources of information on chemicals. There is also the issue of multiple SMILES versions from Daylight Chemical Information Systems. Also, while CAS and Daylight had been around for decades, neither of these organizations had ever made an attempt to have their chemical identifiers embedded in these numerous chemical information resources. Furthermore, the CAS Registry Number is just a number [19] and has no structure information directly associated with it, thus relying on a database with fee-based access. As for SMILES, as pointed out in a recent blog by a chemistry graduate student [20]:

“the limitations of SMILES are difficult to ignore. The same readability that makes SMILES appealing to human eyes limits its scope significantly. The innards of the SMILES algorithm(s) are fairly simple from a chemist’s perspective, and do not take into account spontaneous structural changes like tautomerization (or even the structural equivalence of resonance forms). There are multiple algorithms, meaning there is not, strictly speaking, a one-to-one relationship between structure and SMILES string. Finally, SMILES is a proprietary format whose algorithms are kept under lock and key—with the notable exception of the OpenSMILES project.”

With the majority of commercial organizations initially supporting “institutionalization” of InChI in Europe, and the two major supporters (the Royal Society of Chemistry and Nature) in the UK, it was decided to create a non-for-profit UK charity to take over the development and maintenance of the InChI algorithm and related activities [21]. The InChI Trust's mission and goal is to enable the interlinking and combining of chemical, biological and related information, using unique machine-readable chemical structure representations to facilitate and expedite new scientific discoveries.

While the Trust members are extremely competitive in many ways, and the timing for asking for financial support during a period of prolonged world-wide economic turndown less than ideal, during the three years since the Trust was established in 2009 those involved in managing and operating the project have developed both a tactical and a strategic vision for InChI. The result has been an unusual set of characteristics of the project as listed below:

• Consensus

• Technical competence

• Political and technical cooperation

• Pre-competitive collaboration

• No competition with commercial products

• No mission creep

• IUPAC blessing and rapid IUPAC acceptance

• Understanding of the Internet and how it can be effectively used in linking information associated with chemicals

• Vision of the future

### InChI certification software

For InChI to be a functioning standard assurance is needed that InChIs created anywhere in the world and by anyone using the algorithm for the same structure are the same. In order to make this assurance the project has created an InChI certification software and database product, the InChI Certification Suite. Very simply, the suite is a software package developed and designed to check that an installation of the InChI program has been performed correctly. The certification programs test the installation against a broad set of structures (which are provided with the suite package). Once the programs are run and the results sent back to the Trust, an "InChI Certified" logo is sent to the appropriate person or organization. The InChI Trust certification logo can then be included on the pages of the organization’s web site for all users to see.

The InChI Trust certification suite package is available at no cost to associate and full members of the Trust. All others pay an annual fee for the package, currently US $5,000 [22].

### Introduction to InChI and the InChIKey

Chemists use diagrammatic representations to convey structural information, and these are sometimes supplemented by verbal descriptions of structure. Conventional chemical nomenclature is a means of specifying a chemical structure in words, and systematic nomenclature provides an unambiguous description of a structure, a diagram of which can be reconstructed from its systematic name. The IUPAC International Chemical Identifier (InChI**)** is a machine-readable string of symbols which enables a computer to represent the compound in a completely unequivocal manner. InChIs are produced by computer from structures drawn on-screen, and the original structure can be regenerated from an InChI with appropriate software. An InChI is not directly intelligible to the normal human reader (and was not designed to be readily intelligible); it is more like a bar code.

There is more than one way to specify molecular structures, and those based on ‘connection tables’ (specifications of atomic connectivities) are more suitable for processing by computer than conventional nomenclature, as they are matrix representations of molecular graphs, readily governed and handled by graph theory. This does not imply that traditional IUPAC nomenclature will eventually be displaced by computer methods; the continued development of verbal nomenclature has run in parallel with the development of InChIs.

An InChI is generated from a computerized representation of a molecular structure diagram produced by chemical structure-drawing software. A full and detailed description of the InChI and of the software for its generation is available from the IUPAC website [23], and further information can be obtained from the website of the InChI Trust [24].

A paper giving a full account of the InChI project is in preparation [25]. Commercial structure-drawing software packages that will generate an InChI and the related InChIKey are available from several organizations, which are listed in a Wikipedia article under software and services [26].

While the InChI is a unique string identifying a defined chemical structure, its length increases with the size of the structure drawn. A structure with 100+ atoms (InChI currently allows for up to 1000 atoms) gives a very long string. Trying to use it in any Internet search engine (Google, Bing, Yahoo, and so on) does not produce reliable results. These search engines do not use more than 30+ characters; they do not care about case sensitivity; and they do not care about a number of InChI characters such as -,+, /, or \. Thus, at a seminar two of the authors gave (SRH and SES) at Google [27] it was made very clear that the project needed something more than the InChI string if InChIs were be used for productive and successful internet searches. The simplest and best way to compress a string was to use a well-known, well studied, well understood, and well described hashing algorithm [28]. Thus the InChIKey was created. The InChIKey is a 27-character compacted version of InChI which is intended for Internet and database searching/indexing and is based on an SHA-256 hash of the InChI character string [29].

### Short description of InChI and InChIKey

Other articles in this thematic issue of the Journal of Cheminformatics have further programmer and technical details on the InChI algorithm and related matters; thus the following will only be a brief introduction and description of InChI.

The conversion of structural information to its InChI is based on a set of IUPAC structure conventions and the InChI project’s rules for normalization and canonicalization (conversion to a single, predictable sequence) of a structure representation. The resulting InChI is simply a series of characters that serve to identify uniquely the structure from which it was derived. This conversion of a graphical representation of a chemical substance into the unique InChI character string can be carried out automatically by anyone using the freely available programs, and the facility can be built into any program dealing with chemical structures. The InChI uses a layered format to represent all the available structural information relevant to compound identity. InChI layers are listed below. Each layer in an InChI representation contains a specific type of structural information. These layers, automatically extracted from the input structure, are designed so that each successive layer adds additional detail to the Identifier. The specific layers generated depend on the level of structural detail available and whether or not allowance is made for tautomerism. Of course, if there are any ambiguities or uncertainties in the original structure representation, these will remain in the InChI. Since we know that chemists do not always draw a structure for any particular compound in the same way, 100% perfection will not be possible.

This layered structure design of an InChI offers a number of advantages. If two structures for the same substance are drawn at different levels of detail, the one with the lower level of detail will, in effect, be contained within the other. Specifically, if one substance is drawn with stereo-bonds and the other without, the layers in the latter will be a subset of the former. The same will hold for compounds treated by one author as tautomers and by another as exact structures with all hydrogen atoms fixed. This can work at a finer level. For example, if one author includes a double bond and tetrahedral stereochemistry, but another omits stereochemistry, the InChI for the latter description will be contained within that for the former.

#### The structure of InChIs

The successive layers of an InChI are characterized as follows:

1. Formula

2. Connectivity (no formal bond orders)

a. disconnected metals

b. connected metals

3. Isotopes

4. Stereochemistry

a. double bond

b. tetrahedral

5. Tautomers (on or off)

**Figure 1:**
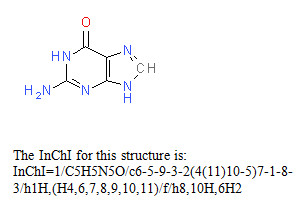
Guanine.

**Figure 2:**
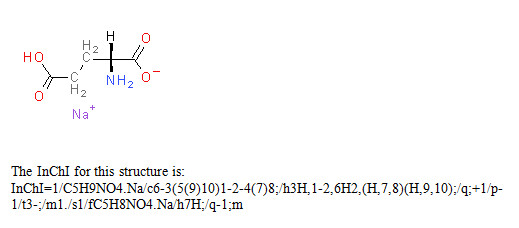
Monosodium glutamate.

Two examples of InChI representations are given belowin Figures 1 and 2. However, it is important to recognize that InChI strings are intended for use by computers and end-users need not understand any of their details. One should think of InChIs like bar codes - very useful and essentially unreadable by a human. In fact, the open nature of InChI and its flexibility of representation, after implementation into software systems, may allow chemists to be even less concerned with the details of structure representation by computers.

The layers in the InChI string are separated by the slash, /, followed by a lower-case letter (except for the first layer, the chemical formula), with the layers arranged in a predefined order.

In the Examples above the following segments are included:

InChI version number

/chemical formula

/c connectivity-1.1 (excluding terminal H)

/h connectivity-1.2 (locations of terminal H, including mobile H attachment points)

/q charge

/p proton balance

/t tetrahedral parity

/m parity inverted to obtain relative stereo (1 = inverted, 0 = not inverted)

/s stereo type (1 = absolute, 2 = relative, 3 = racemic)

/f chemical formula of the fixed-H structure if it is different

/h connectivity-2 (locations of fixed mobile H)

One of the most important applications of InChI is the facility to locate mention of a chemical substance using internet-based search engines. This is made easier by using the InChIKey. As noted previously, the InChIKey is a 27-character representation that, because it is compressed, cannot be reconverted into the original structure, but it is not subject to the undesirable and unpredictable breaking of longer character strings by some search engines. The usefulness of the InChIKey as a search tool is enhanced if it is derived from a ‘standard’ InChI, i.e., an InChI produced with standard option settings for features such as tautomerism and stereochemistry.

**Figure 3:**
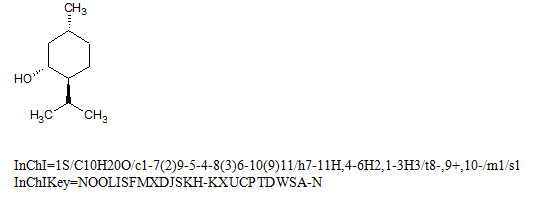
(-)-Menthol.

In Figure 3, the standard InChI is denoted by the letter S after the version number. Use of the InChIKey also allows searches based solely on atomic connectivity (first 14 characters). The software for generating InChIKey is also available from the InChI Trust website [26].

First block of InChIKey, 14 letters, encodes molecular skeleton (connectivity; in this case, up to ‘/t’ marker).

Second block, 8 letters, encodes stereochemistry (three tetrahedral stereogenic centers) and isotopes (not present in the structure), S indicates standard InChIKey produced from standard InChI.

A indicates InChI version 1; last character indicates the number of protons, N meaning neutral.

The first block of InChIKey encodes molecular skeleton while the second block represents various kinds of isomerism (stereo, tautomeric, etc.). The InChIKey is designed to be a nearly unique substitute for the parent InChI. However, a single InChIKey may occasionally map to two or more InChI strings (collision). The appearance of collision itself does not compromise the signature as collision-free hashing is impossible; the only viable approach is to set and keep a reasonable level of collision resistance which is sufficient for typical applications. This was proved by computational experiments described in a dedicated paper published in this journal issue [30].

“How many miles have we gone, InChI by InChI” is the title of a summary of the April 2012 ACS CINF symposium on InChI [31]. While this summary was rather positive in what it said about InChI, we feel it necessary and useful to also provide commentary on the problems and flaws with the current InChI. We do this for the purpose of being scientifically honest, and also to indicate we know some, if not most, of what needs to be in the next version (version 2) of InChI. In other words we know we must continue to move forward. We also know that while there will some changes to InChIs created in version 2, these will be few and far between. And in some cases with relatively simple straightforward organic molecules there will be no change. We know of no structure system devised to date that does not contain errors or flaws, which are later discovered and corrected. One very positive aspect of the InChI project has been that because so many people are using the algorithm and so many different structures are being processed we have been able to find problems and errors sooner than if we worked in a vacuum with only our own inertial databases. The Sourceforge InChI-discuss list-server with some 100 subscribers has been invaluable to the project in finding and dealing with issues [32]. In other words, crowd sourcing is great and we are finding that needle in a haystack with the help of hundreds of people around the world.

**Figure 4:**
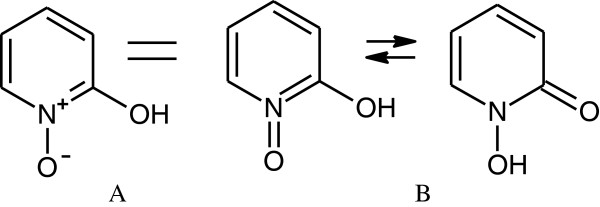
1,4-oxime/nitroso tautomerism.

While going from drawn structure to an InChI and/or InChIKey works 100% of the time when that class of chemicals has been programmed into the algorithm (and does not generate an InChI when the program does not have the capability for that class of structures), the reverse process is not 100% reliable. We estimate that converting an InChI to its structure works more than 99% of the time. If one uses the AuxInfo layer capability available within the InChI algorithm, conversion from InChI to structure works 100% of the time. Another current flaw in version 1 of the InChI algorithm which is observed on very rare occasions is that more than one structure will generate the same InChI. And, due to the variability in chemist’s training it is possible for two differently drawn structures of the same compound to generate two different InChIs, as seen in Figure 4.

Currently the InChI algorithm generates two different InChIs for the above structure depending on how it is drawn:

A. pyridin-2-ol *N*-oxide

InChI=1S/C5H5NO2/c7-5-3-1-2-4-6[5]8/h1-4,7H

B. *N*-hydroxypyridin-2-(1*H*)-one

InChI=1S/C5H5NO2/c7-5-3-1-2-4-6[5]8/h1-4,8H

It is expected that this will be resolved in InChI version 2, which is currently being considered by the IUPAC InChI subcommittee. It is certainly a fair question to ask why these problems exist and why they were not addressed in the original work when the algorithm was first developed. The reason, which is common in almost every project, and especially in a project like this in which we are working to create a standard, is simple. While we wanted to believe we would reach our goal of creating a standard that the world would accept, we knew full well the odds were not in our favor. Hence, the initial goal of 99%, not 100%. We believe the project has already been a major success, with success defined as un-coerced adoption, since adoption must be a bottom-up process.

The enormous databases compiled by organizations such as PubChem [33], the US National Cancer Institute [34], and ChemSpider [35] contain millions of InChIs and InChIKeys, which allow sophisticated searching of these collections. PubChem provides InChI-based structure-search facilities (for both identical and similar structures), and ChemSpider offers both search facilities and web services enabling a variety of InChI and InChIKey conversions [36]. The NCI Chemical Structure Lookup Service provides InChI-based search access to over 100 million chemical structures from over 80 different public and commercial data sources.

### Summary

InChI is almost complete for individual ‘defined’ structures. It has been able to cover a sufficient area of chemistry to be of extreme value and use to the international chemical and related scientific communities. Even though InChI is not perfect, and will probably never be [37], the past, current, and future efforts will make it the worldwide chemical structure representation standard for linking information on chemicals from databases and resources around the world. At this time it seems clear that the goal to enable interlinking and combining of chemical, biological and related information, using the unique InChI machine-readable chemical structure representations to facilitate and expedite new scientific discoveries is eminently achievable.

## Competing interests

The authors declare that they have no competing interests.

## Authors’ contributions

SH and SS originated the project. SH and AM led the IUPAC activities including the establishment of the IUPAC InChI subcommittee and the InChI Trust. DT and IP did the programming. All authors read and approved the final manuscript.

## References

[B1] Wiswesser WJ 107 Years of Line-Formula Notations (1861–1968) J Chem Doc 1968 8 116 http://en.wikipedia.org/wiki/Structural_formula c) John M. Barnard, “Structure Representation”, chapter in Encyclopedia of Computational Chemistry, 5 Volume Set Paul von Ragué Schleyer (Editor-in-Chief) ISBN: 0-471-96588-X, John Wiley,1999

[B2] Weininger D SMILES, a Chemical Language and Information System. 1. Introduction to Methodology and Encoding Rules J Chem Inf Comput Sci 1988 28 1 31 36 SMILES is available from Daylight Chemical Information Systems. PO Box 7737, Laguna Niguel, CA 92677. http://www.daylight.com/10.1021/ci00057a005

[B3] CAS Registry J Chem Inf Comput Sci 1978 18 1 58 http://www.cas.org/content/chemical-substances 10.1021/ci60013a609

[B4] Dalby A Nourse JG Hounshell WD Gushurst AKI Grier DL Leland BA Laufer J Description of several chemical structure file formats used by computer programs developed at Molecular Design Limited J Chem Inf Comput Sci 1992 32 244 255 http://accelrys.com/products/informatics/cheminformatics/ctfile-formats/no-fee.php 10.1021/ci00007a012

[B5] http://www.cas.org/content/chemical-substances

[B6] a) http://en.wikipedia.org/wiki/Black_swan_theory b) http://en.wikipedia.org/wiki/The_Black_Swan_%28Taleb_book%29

[B7] In particular the Mass Spectral Libraryhttp://www.nist.gov/srd/, http://www.nist.gov/srd/nist1a.cfm, a database originally started by one of the authors (SRH) and later transferred to NIST for enhancements, enlargement and distribution

[B8] http://old.iupac.org/news/archives/2000/NRT_Report.html, http://www.iupac.org/fileadmin/website/news/2000/NRT_Report.pdf

[B9] Town B Kilmorie Consulting 2000 24A Elsinore Road, London SE23 2SL, UK bill.town@kilmorie.com

[B10] The project was officially announced in Chemistry International, 23(3), May 2001 http://www.iupac.org/publications/ci/2001/may/project_2000-025-1-050.html

[B11] IUPAC InChI project information is available at: http://www.iupac.org/projects/2000/2000-025-1-800.html

[B12] The slides from these two presentations are available at: http://www.hellers.com/steve/pub-talks/

[B13] David A Chemists Synthesize a Single Naming System Nature 2002 417 369 12024181 10.1038/417369a

[B14] Michael F Unique Label for Compounds C&E News 2002 80 48 33 Also available on the web at: http://pubs.acs.org/isubscribe/journals/cen/80/i48/html/8048sci1.html

[B15] http://www.iupac.org/home/publications/e-resources/inchi.html

[B16] http://www.iupac.org/nc/home/about/members-and-committees/db/division-committee.html?tx_wfqbe_pi1[title]=Chemical%20Nomenclature%20and%20Structure%20Representation%20Division&tx_wfqbe_pi1[publicid]=800

[B17] http://en.wikipedia.org/wiki/List_of_patent_claim_types

[B18] http://www.nist.gov/pml/wmd/metric/metric-policy.cfm and http://en.wikipedia.org/wiki/Metric_Act_of_1866

[B19] CAS REGISTRY and CAS Registry Number FAQshttp://www.cas.org/content/chemical-substances/faqs

[B20] http://graphiteworks.wordpress.com/2012/08/02/chemoinformatics-curiosities-a-chemical-educators-perspective-on-inchi/#comment-1497

[B21] http://inchi-trust.org/index.php?q=node/6

[B22] http://www.inchi-trust.org/inchi-certification-suite/

[B23] http://www.iupac.org/nc/home/publications/e-resources/inchi/download.html?sword_list[]=inchi

[B24] http://www.inchi-trust.org/downloads/ and http://www.inchi-trust.org/faq/

[B25] Igor P J Chemoinformatics 2012

[B26] http://en.wikipedia.org/wiki/International_Chemical_Identifier

[B27] http://www.hellers.com/steve/pub-talks/google-1007/frame.htm

[B28] http://en.wikipedia.org/wiki/Hash_function

[B29] http://en.wikipedia.org/wiki/SHA-2

[B30] Andrey E Alan MN Kirill B Dmitrii T Steve H InChIKey collision resistance: an experimental testing Igor Pletnev J Cheminformatics 2012 4 39 10.1186/1758-2946-4-39 PMC3558395 23256896

[B31] http://bulletin.acscinf.org/node/325

[B32] http://inchi.sourceforge.net/ and https://lists.sourceforge.net/lists/listinfo/inchi-discuss

[B33] http://pubchem.ncbi.nlm.nih.gov

[B34] http://cactus.nci.nih.gov/cgi-bin/lookup/search

[B35] http://www.chemspider.com/

[B36] http://www.chemspider.com/InChI.asmx

[B37] InChI is the worst computer readable structure representation except for all those other forms that have been tried from time to time. With apologies to Sir Winston Churchill (House of Commons speech on Nov. 11, 1947)

